# Effect of Nebivolol on MIBG Parameters and Exercise in Heart Failure with Normal Ejection Fraction

**DOI:** 10.5935/abc.20160046

**Published:** 2016-05

**Authors:** Leandro Rocha Messias, Aryanne Guimarães Ferreira, Sandra Marina Ribeiro de Miranda, José Antônio Caldas Teixeira, Jader Cunha de Azevedo, Ana Carolina Nader Vasconcelos Messias, Elisabeth Maróstica, Claudio Tinoco Mesquita

**Affiliations:** 1 Universidade Federal Fluminense, Rio de Janeiro, RJ - Brazil; 2 Hospital Procardíaco, Rio de Janeiro, RJ - Brazil; 3 Hospital Federal dos Servidores do Estado do Rio de Janeiro, Rio de Janeiro, RJ - Brazil

**Keywords:** Heart failure, exercise testing, MIBG, nebivolol

## Abstract

**Background:**

More than 50% of the patients with heart failure have normal ejection fraction (HFNEF). Iodine-123 metaiodobenzylguanidine (123I-MIBG) scintigraphy and cardiopulmonary exercise test (CPET) are prognostic markers in HFNEF. Nebivolol is a beta-blocker with vasodilating properties.

**Objectives:**

To evaluate the impact of nebivolol therapy on CPET and123I-MIBG scintigraphic parameters in patients with HFNEF.

**Methods:**

Twenty-five patients underwent 123I-MIBG scintigraphy to determine the washout rate and early and late heart-to-mediastinum ratios. During the CPET, we analyzed the systolic blood pressure (SBP) response, heart rate (HR) during effort and recovery (HRR), and oxygen uptake (VO_2_). After the initial evaluation, we divided our cohort into control and intervention groups. We then started nebivolol and repeated the tests after 3 months.

**Results:**

After treatment, the intervention group showed improvement in rest SBP (149 mmHg [143.5-171 mmHg] versus 135 mmHg [125-151 mmHg, p = 0.016]), rest HR (78 bpm [65.5-84 bpm] versus 64.5 bpm [57.5-75.5 bpm, p = 0.028]), peak SBP (235 mmHg [216.5-249 mmHg] versus 198 mmHg [191-220.5 mmHg], p = 0.001), peak HR (124.5 bpm [115-142 bpm] versus 115 bpm [103.7-124 bpm], p= 0.043), HRR on the 1st minute (6.5 bpm [4.75-12.75 bpm] versus 14.5 bpm [6.7-22 bpm], p = 0.025) and HRR on the 2nd minute (15.5 bpm [13-21.75 bpm] versus 23.5 bpm [16-31.7 bpm], p = 0.005), but no change in peak VO_2_ and 123I-MIBG scintigraphic parameters.

**Conclusion:**

Despite a better control in SBP, HR during rest and exercise, and improvement in HRR, nebivolol failed to show a positive effect on peak VO2 and 123I-MIBG scintigraphic parameters. The lack of effect on adrenergic activity may be the cause of the lack of effect on functional capacity.

## Introduction

Approximately 50% of the patients hospitalized with heart failure (HF) have normal ejection fraction (HFNEF).^1^ Compared with patients with HF with reduced ejection fraction (HFREF), those with HFNEF have a few different characteristics such as a higher frequency in women, elderly, and diabetics, and a greater prevalence of atrial fibrillation, obesity, and hypertension.^2,3^

Nebivolol, a 3rd generation beta-1-selective beta-blocker with vasodilating properties mediated by L-arginine/nitric oxide (NO), is associated with improvement in endothelial function^4^ and evidence of improvement in diastolic function.^5^ Results from the SENIORS^6^ study have shown that nebivolol is well tolerated by elderly patients with HF and has similar effects in both HFREF and HFNEF.

Cardiac imaging with metaiodobenzylguanidine labeled with iodine 123 (123I-MIBG) is a noninvasive method in nuclear medicine to evaluate the adrenergic activity and sympathetic innervation of the heart, including the uptake, reuptake, storage, and release of noradrenaline in presynaptic nerve terminals.^7,8^ The early heart-to-mediastinum (H/M) ratio evaluates the integrity of the sympathetic nerve terminal, whereas the late H/M ratio evaluates its physiology.^7^ The washout (WR) rate assesses the degree of adrenergic activity.^7^ According to some studies, ^123^I-MIBG scintigraphic parameters are prognostic markers in HFNEF.^9,10^

Cardiopulmonary exercise test (CPET) may be used in HF to detect ischemia^11^ and assess symptoms,^11^ chronotropic response,^12-14^ heart rate (HR) during recovery (HRR),^15^ and functional capacity (FC).^15,16^ Patients with HFNEF may have chronotropic incompetence,^12,17^ low FC,^12,17^ increase in the minute ventilation to carbon dioxide output (VE/VCO_2_) slope^18^ and inadequate HRR response.^12^ These findings are similar to those in HFREF,^14,15^ but their physiopathology has not been entirely clarified.

Based on the limited knowledge about the effect of beta-blocker therapy on the cardiac adrenergic function in HFNEF, we designed this study to assess if nebivolol would modify, in the short-term, the abnormalities in cardiac sympathetic function and affect the FC and other exercise variables positively.

## Methods

We conducted a prospective study with 25 consecutive patients attending our HF clinic. The inclusion criteria were: age > 18 years, signs and symptoms of HF,^16^ left ventricular ejection fraction (LVEF) ≥ 50% with echocardiographic evidence of diastolic dysfunction,^2^ in addition to the patient's consent on a signed consent form. We excluded patients with diabetes, atrial fibrillation, pacemaker, or any other contraindication to CPET. The project was approved by the Ethics Committee at our institution.

To classify the HF according to its etiology, we used the following criteria: ischemic (previous infarction, inactive area detected by electrocardiography, or coronary cineangiography showing a left coronary trunk lesion ≥ 50% or a ≥ 70% lesion in one of the three main systems),^19^ hypertensive (history of hypertension and absence of criteria of ischemic HF), and others (including patients who were not classified as ischemic or hypertensive).

In patients without criteria for ischemic HF but with ischemic manifestations during the CPET, we expanded the investigation with myocardial perfusion scintigraphy and coronary cineangiography, if necessary, to evaluate the occurrence of coronary artery disease. If the patient showed no signs of exercise-induced myocardial ischemia, we then maintained the etiological classification as nonischemic.

All patients underwent 123I-MIBG scintigraphy and CPET. After this initial phase, we divided the sample into two groups: the first 14 volunteers received treatment with nebivolol (nebivolol group) and the last 11 volunteers composed the control group. We started the treatment with nebivolol at the dose of 1.25 mg/day with weekly dose increases (doubling the previous dose), aiming to achieve a target dose of 10 mg/day, or an HR between 50-60 bpm, or a systolic blood pressure (SBP) between 90-100 mmHg.^6^ If the patient was already using another beta-blocker, we suspended this beta-blocker and started nebivolol following the same described protocol. After 3 months of therapeutic optimization, we repeated the evaluations with 123I-MIBG scintigraphy and CPET.

The purpose of the 123I-MIBG scintigraphy was to evaluate the integrity of the sympathetic nerve terminal through quantification of early (30 min after injection of the radiotracer) and late H/M (4 h after the injection) ratios by anterior planar image of the thorax.^7^ The sympathetic activity was estimated with the WR rate, calculated with the formula:^7,9^ WR (%) = (H - M) 30 min - (H - M) 4 h x 100 / (H - M) 30 min. All scintigraphic tests were performed on a Siemens® digital tomographic Anger-like scintillation camera (Single Photon Emission Computed Tomography), model E-cam with dual detector and low-energy and high-resolution collimator.

The CPET was symptom-limited and conducted on a Centurion 300® treadmill using an individualized ramp protocol for better evaluation of the kinetics of oxygen uptake (VO_2_).^11,20^ We started the test at a speed of 1.6 km/h, individualized the exercise to obtain an effort duration of 8-12 minutes, and conducted an active recovery at a speed of 1.6 km/h during the first 2 minutes and passive recovery in the orthostatic position for an additional 6 minutes. We used the software Ergo PC Elite version 13/2.2 (Micromed®).

To evaluate the respiratory gases, we used the metabolic analyzer MedGraphics® VO2000. Using a medium-flow pneumotachograph, we measured a gas sample every 10 seconds using a mask for patient-equipment adaptation. The peak VO_2_ was defined as the highest VO_2_ measured during the last 30 seconds of the exercise.^20^ To determine the VO_2_ in the anaerobic threshold, we used the ventilatory equivalents method.^20^ The VE/VCO_2_ slope was calculated with the inclination model of the software.^15,20^

We measured the HR using the R-R interval at rest, peak effort, and recovery. We analyzed the chronotropic response with the chronotropic response index (CRI):^14^ CRI (%) = (peak HR - rest HR) x 100 / (220 - age - rest HR). HRR was determined at the 1st and 2nd minutes by subtracting the peak HR by the HRR.^12,15^ Blood pressure was measured with a mercury sphygmomanometer (Wan Ross®). We evaluated the SBP at rest and peak effort, and the variation during the effort (peak SBP - rest SBP).^21^

We conducted a pilot study to calculate the sample size. According to the obtained data, nine patients would be required per group for a β error of 80% and an α error of 5%. The sample power calculated at the end of the study showed that 25 patients met a statistical power of 80% to identify 12.8% of difference in peak SBP.

Our data had a nonparametric distribution and are presented as median/interquartile range when the variables are quantitative and percentage when they are qualitative. The statistical analysis was performed with the software SPSS*,* version 15. We used the chi-square test to compare qualitative variables and the Mann-Whitney U test to compare quantitative variables in a first analysis between the control and intervention groups before the intervention. In a second analysis, we used the paired Wilcoxon test to compare the values at baseline with those obtained at 3 months in the control group and the values at baseline with those obtained 3 months after the intervention with nebivolol in the intervention group. We considered a p value < 0.05 as significant.

## Results

Table 1 shows the clinical characteristics, echocardiographic parameters, and medications used by the participants. There were no significant differences in the variables age, gender, and body mass index (BMI), or in echocardiographic parameters. All patients were hypertensive and showed no significant differences in the incidence of dyslipidemia, smoking, or in the etiology of the HF. Most patients were in New York Heart Association (NYHA) functional classes II and III. There were no significant differences in the medications used by the participants.

**Table 1:** Baseline characteristics of the cohort

**Variable**	**Intervention**	**Control**	**p**
n = 25	14	11	-
Age (years)	56.5 (50.75 - 62.25)	61 (52 - 71)	0.291*
Gender %	-	-	0.452†
Female	71.42	81.81	-
Male	28.58	18.19	-
BMI (kg/m^2^)	31.51 (26.62 - 34.77)	33.32 (26.34 - 37.18)	0.647*
Hypertension %	100	100	1†
Dysllpldemla %	71.42	72.72	0.649†
Smoking %	35.71	18.18	0.305†
Etlology %	-	-	0.697†
Ischemic	7.14	9.09	-
Hypertensive	92.86	90.91	-
Others	0	0	-
Echocardiography	-	-	-
LVEF %	63.5 (60.75 - 72.25)	67 (54 - 71)	0.979*
E/E'	16.15 (15.35 - 17.25)	15.2 (13.88 - 16.9)	0.183*
E/A	0.41 (0.32 - 0.74)	0.38 (0.22 - 0.5)	0.244*
LAVI (ml/m^2^)	45.26 (41.98 - 48.72)	40.58 (36.6 - 45.54)	0.107*
LVMI (g/m^2^)	124,05(113,5-131,35)	124 (97.36 - 130)	0.609*
FC /NYHA%	-	-	0.444†
I	7.15	18.18	-
II	42.85	54.54	-
III	50	27.28	-
IV	0	0	-
Medications in use %	-	-	-
Beta-blocker	42.85	63.63	0.265†
Atenolol	66.66	42.85	-
Carvedilol	33.34	42.85	-
Propranolol	0	14.3	-
ACEI/ARA II	85.71	81.81	0.604†
Hydralazine	14.28	18.18	0.604†
Nitrate	14.28	36.36	0.209†
Spironolactone	14.28	27.27	0.378†
Diuretic	71.42	54.54	0.325†
Ca channel blocker	64.28	36.36	0.163†
Clonidine	42.85	27.27	0.352†
Aspirin	28.57	45.45	0.325†
Statin	35.71	63.63	0.163†

* Mann-Whitney U Test;

† Chi-square test; N: number of patients; BMI: body mass index; LVEF: left ventricular ejection fraction; E/E’: ratio of the mitral peak velocity of early filling to the early diastolic mitral annular velocity; E/A: ratio of the mitral peak velocity of early filling to the mitral peak velocity of late filling; LAVI: left atrial volume index; LVMI: left ventricular mass index; FC: functional class; NYHA: New York Heart Association; ACEI: angiotensin II converting enzyme inhibitor; AAR II: angiotensin II receptor antagonist; Ca: calcium.

The CPET and 123I-MIBG scintigraphic variables are shown in Table 2. On initial analysis, we observed that there were no significant differences in the CPET variables. Both groups started the test hypertensive and responded to the effort with hypertension,^11^ chronotropic incompetence,^14^ low FC,11,18,20 and oxygen pulse (O_2_) below the expected level,^11^ but had a good prognosis according to the VE/VCO slope.^22^ According to the median respiratory coefficient (R), all patients performed a maximum test (R > 1.05)^23^ and managed to reach the anaerobic threshold, demonstrating that the CPET was adequate.^20^ The intervention group (the group which was later allocated to nebivolol) presented a worse HRR in the 1st and 2nd minutes, but the differences were not significant. The control group had lower median early and late H/M ratios and 123I-MIBG WR, but these results were also not significantly different.

**Table 2:** Comparison of CPET and 123I-MIBG scintigraphic variables

**Variable**	**Intervention**	**Control**	**p**
ISBP mmHg	149 (143.5 - 171)	162 (132 - 170)	0.851
IDBP mmHg	91 (80.5 - 106.5)	90 (78 - 104)	0.727
IHR bpm	78 (65.5 - 84)	66 (55 - 72)	0.066
PSBP mmHg	235 (216.5 - 249)	230 (216 - 238)	0.467
PDBP mmHg	111 (102.5 - 120)	104 (82 - 110)	0.12
PHR bpm	124.5 (115 - 142.75)	117 (104 - 146)	0.501
SBPDE mmHg	69 (52 - 102.5)	76 (52 - 96)	0.647
CRI%	60.11 (43.59 - 81.57)	58.7 (40.74 - 91.36)	0.851
HRRIst bpm	6.5 (4.75 - 12.75)	18 (7 - 21)	0.085
HRR2nd bpm	15.5 (13 - 21.75)	26 (19 -33)	0.058
VO_2_ AT ml.(kg.min)^-1^	10.89 (7.97 - 12.58)	10.62 (7.89 - 14.29)	0.886
Percent VO_2_ peak at AT %	72.9 (66.6 - 86.4)	77.7 (72.52 - 85.12)	0.508
R	1.10 (1.03 - 1.16)	1.18 (1.07 - 1.23)	0.202
Peak VO_2_ ml.(kg.min)^-1^	14.07 (10.71 - 18.03)	12.75 (8.48 - 16.77)	0.851
VE/VCO_2_ slope	22.73 (20.02 - 26.61)	23.37 (22.53 - 26.9)	0.467
O_2_ pulse ml.(kg.min)^-1^/bpm	8.6 (7.12 - 11.6)	9 (6.6 - 10.8)	0.893
Percent O_2_ pulse predicted %	61.2 (41.75 - 83.17)	60.8 (45.4 - 85.1)	0.893
H/M30min	1.89 (1.65 - 1.97)	1.6 (1.56 - 1.8)	0.134
H/M4h	1.77 (1.57 - 1.94)	1.58 (1.22 - 2)	0.344
WR%	29.5 (21.85 - 51)	27 (14.3 - 30)	0.222

ISBP: initial systolic blood pressure; IDBP: initial diastolic blood pressure; IHR: initial heart rate; PSBP: systolic blood pressure at peak effort; PDBP: diastolic blood pressure at peak effort; PHR: heart rate at peak effort; SBPDE: systolic blood pressure variation during effort; CRI: chronotropic reserve index; HRR1st: heart rate variation at the first minute of recovery; HRR2nd: heart rate variation at the second minute of recovery; VO_2_: oxygen uptake; AT: anaerobic threshold; R: respiratory coefficient; VE/VCO_2_ slope: minute ventilation to carbon dioxide output slope; O_2_: oxygen; H/M30min: heart to mediastinum ratio 30 minutes after injection of the radiotracer (early); H/M4h: heart to mediastinum ratio 4 hours after injection of the radiotracer (late); WR: washout rate.

After this initial evaluation, we started the treatment with nebivolol in the intervention group. The average administered dose of nebivolol was 9.29 ± 1.81 mg/day. After 3 months, we repeated the CPET and 123I-MIBG scintigraphy and compared the results in each group with their respective baseline results (Table 3).

**Table 3:** Comparison of cardiopulmonary exercise test and 123I-MIBG scintigraphic variables after treatment with nebivolol

	**Intervention**			**Control**		
**Variable**	**Baseline**	**3 months**	**p**	**Baseline**	**3 months**	**p**
ISBP mmHg	149(143.5-171)	135(125-151)	0.016	162 (132 -170)	148 (132-160)	0.213
IDBP mmHg	91 (80.5-106.5)	91(87.5-107.5)	0.179	90 (78 - 104)	100 (70-102)	0.682
IHR bpm	78 (65.5 - 84)	64.5(57.5-75.5)	0.028	66 (55 - 72)	64 (61-77)	0.656
PSBP mmHg	235(216.5-249)	198(191-220.5)	0.001	230 (216 -238)	222 (210-240)	0.683
PDBP mmHg	111(102.5-120)	113 (91.5-118)	0.441	104 (82 - 110)	110(78-120)	0.24
PHR bpm	124.5(115-142)	115(103.7-124)	0.043	117 (104 -146)	123(106-138)	0.919
SBPDE mmHg	69 (52 - 102.5)	69 (38-86)	0.116	76 (52 - 96)	72 (60-108)	0.447
CRI %	60.1(43.5-81.5)	51.5(32.9-70.5)	0.124	58.7(40.7-91.3)	65.2(40.2-89.2)	0.929
HRR 1st bpm	6.5(4.75-12.75)	14.5(6.7-22)	0.025	18 (7 - 21)	18 (11-29)	0.285
HRR 2° bpm	15.5(13- 21.75)	23.5(16-31.7)	0.005	26 (19 -33)	23 (14-41)	0.54
VO_2_ AT ml.(kgml)^-1^	10.89(7.9-12.5)	10.5(7.8-13.6)	0.917	10.6(7.8-14.2)	9.8(5.9-13.5)	0.169
Percent O_2_ peak at AT %	72.9(66.6-86.4)	78.1(65.5-90.6)	0.422	77.7(72.5-85.1)	77.4(65.3-82)	0.333
R	1.1(1.03 - 1.16)	1.16(1.02-1.35)	0.158	1.18(1.07-1.23)	1.25(1.1-1.4)	0.203
Peak VO_2_ ml.(kgml)^-1^	14.07(10.7- 18)	14.18(9.3-17.1)	0.551	12.75(8.4-16.7)	13.02(7.4-17.8)	0.155
VE/VCO_2_ slope	22.73(20-26.6)	21.7(19.3-28.8)	0.363	23.3(22.5-26.9)	22.5(20.6-27.4)	0.999
O_2_ pulse ml.(kgml)^-1^/bpm	8.6(7.12 - 11.6)	8,9(7.1-12.2)	0.421	9 (6.6 - 10.8)	8.1 (6.1-10.2)	0.005
Percent O_2_ pulse predicted %	61.2(41.7-83.1)	65.1(46.8-80.6)	0.49	60.8(45.4-85.1)	63.6(43.5-84.6)	0.131
H/M30min	1.89(1.65-1.97)	1.85(1.61-1.97)	0.73	1.6 (1.56 - 1.8)	1.63(1.47-1.77)	0.398
H/M 4 h	1.77(1.57-1.94)	1.68(1.58-1.88)	0.263	1.58 (1.22 - 2)	1.52(1.45-1.8)	0.423
WR(%)	29.5(21.85-51)	31(28.2-35)	0.9	27 (14.3 - 30)	30 (15 - 42)	0.722

ISBP: initial systolic blood pressure; IDBP: initial diastolic blood pressure; IHR: initial heart rate; PSBP: systolic blood pressure at peak effort; PDBP: diastolic blood pressure at peak effort; PHR: heart rate at peak effort; SBPDE: systolic blood pressure variation during effort; CRI: chronotropic reserve index; HRR1st: heart rate variation at the first minute of recovery; HRR2nd: heart rate variation at the second minute of recovery; VO_2_: oxygen uptake; AT: anaerobic threshold; R: respiratory coefficient; VE/VCO_2_ slope: minute ventilation to carbon dioxide output slope; O_2_: oxygen; H/M30min: heart to mediastinum ratio 30 minutes after injection of the radiotracer (early); H/M4h: Heart to mediastinum ratio 4 hours after injection of the radiotracer (late); WR: washout rate.

**Figure 1:**
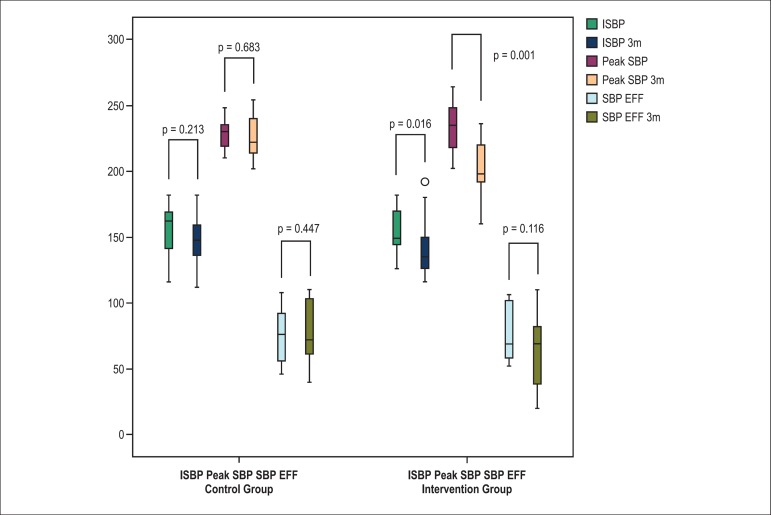
Comparison of blood pressure responses during exercise. ISBP: initial systolic blood pressure; Peak SBP: systolic blood pressure at peak effort; SBP EFF: systolic blood pressure variation during effort; 3m: 3 months.

**Figure 2:**
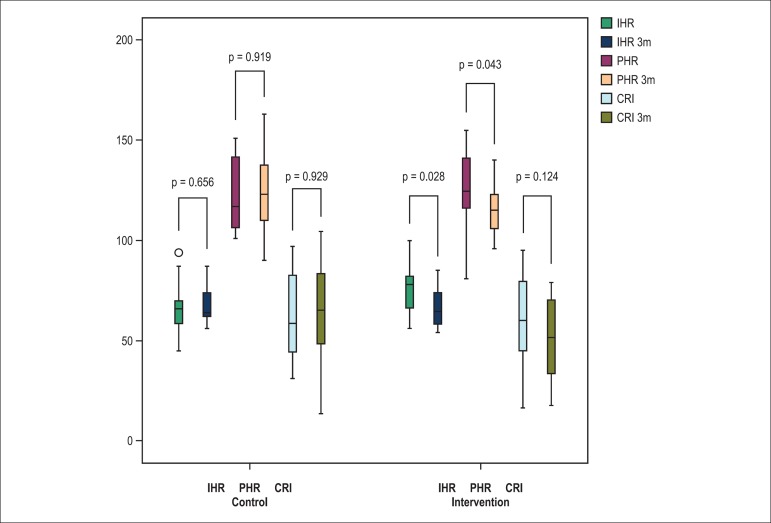
Comparison of heart rate responses during exercise. IHR: Initial heart rate; Peak HR: heart rate at peak effort; CRI: chronotropic response index; 3m: 3 months.

The nebivolol group presented better control in SBP and HR at rest and peak effort but had no significant differences in SBP variation during effort and CRI. Figures 1 and 2 illustrate the patterns of SBP and HR. Patients treated with nebivolol also showed improvement in HRR in the 1^st^ and 2^nd^ minutes. However, nebivolol showed no positive impact on VO_2_ and 123I-MIBG scintigraphic variables, *i.e.*, the therapy was ineffective in improving the FC and the abnormalities in cardiac adrenergic activity.

## Discussion

After 3 months of treatment, nebivolol failed to achieve a positive effect on innervation and cardiac adrenergic activity parameters, detected with 123I-MIBG, or on peak VO_2_ and VE/VCO_2_ slope, even though it led to better control in SBP and HR at rest and peak effort in association with an improvement in HRR.

According to Katoh et al.,^9^ as the deterioration in NYHA functional class, there is a decrease in late H/M ratio and increase in MIBG WR rate. These parameters were associated with a worse prognosis in HFNEF, including increased rates of adverse events associated with a WR rate greater than 26.5%.^9^ In our study, both the nebivolol and control groups presented a WR rate greater than the cutoff point in the study of Katoh et al.,^9^ suggesting a more reserved prognosis in our cohort in general, in addition to an inefficacy of nebivolol to improve the WR rate and the H/M ratio. The lack of a positive impact in 123I-MIBG scintigraphic variables indicates that the drug was unable to act consistently on the adrenergic hyperactivity since clinically effective therapies are consistently associated with improvements in 123I-MIBG scintigraphic parameters in HFREF.^24^ Since our results showed no positive effect on scintigraphic parameters, we can infer that the therapy with nebivolol had no impact on the adrenergic hyperactivity, one of the physiopathologic pathways in HF.^25^

Sugiura et al.^10^ evaluated the 123I-MIBG scintigraphic parameters in HFNEF and demonstrated that the adrenergic activity increases proportionally to the HF severity. These authors have also reported a correlation between the WR rate with the NYHA functional class, FC (assessed with the Specific Activity Scale) and neurohumoral markers,^10^ in addition to a correlation between the WR rate and H/M ratio

with the ratio of the mitral peak velocity of early filling to the mitral peak velocity of late filling (E/A), evaluated with the transmitral flow, suggesting an association between diastolic dysfunction and cardiac adrenergic activity.^10^ In our study, we sought to assess the impact of the therapy with nebivolol on CPET and 123I-MIBG scintigraphic parameters, but even with better control in SBP and HR, nebivolol failed to improve the FC and the cardiac adrenergic activity.

The ADMIRE-HF^26^ study has validated the 123I-MIBG scintigraphy as a prognostic marker in HFREF, demonstrating that this method is able to quantify the cardiac adrenergic innervation. In agreement with the findings by Kato et al.^9^ and Sugiura et al.,^10^ the test may be used to assess patients with HFNEF.

Phan et al.^12^ observed that patients with HFNEF show a lower HR at peak effort, worse chronotropic reserve during exercise, and an inadequate HRR in the 1st minute. The authors^12^ attributed the low FC in HFNEF to chronotropic incompetence. Borlaug et al.^17^ observed that the functional limitation in patients with HFNEF cannot be attributed exclusively to abnormalities in diastolic function^17^ and described as limiting factors for the exercise the chronotropic incompetence, an abnormal vasodilating response, and lower cardiac output during exercise^17^. In another study, Dhakal et al.^27^ reported that patients with HFNEF present abnormal peripheral O_2_ uptake, another limiting factor of VO_2_.

Since the abnormal HR response to exercise is due to changes in the autonomic nervous system, we can affirm that patients with HFNEF have autonomic dysfunction.^28^ This fact can be attributed to an abnormal arterial baroreflex.^17^ It may be possible that patients with HFNEF reach their maximum contractile reserve at an earlier stage of the exercise due to refractoriness to sympathetic stimulation, rather than ineffective stimulation.^14,17^ Since the chronotropic incompetence would be a limiting factor for the exercise, the therapy with nebivolol would not be suitable for its beta-blocking purpose.^29^ However, the positive effect of beta-blocker therapy on the 123I-MIBG scintigraphic parameters in HFREF^30^ could improve the FC.^30,31^. Our group^32^ evaluated patients with HREF and observed that those with a low WR rate, even while on beta-blocker, presented a better FC and chronotropic response when compared with patients with a high WR rate. The current literature has limited data about beta-blocker therapy in HFNEF. In the present study, we did not observe a significant worsening in CRI to justify completely the lack of effect of nebivolol on VO_2_.

Another limiting factor of FC in HFNEF would be an impaired vasodilating reserve that could lead to reduced cardiac output during exercise and reduced muscle perfusion.^17^ The vasodilating reserve is impaired in part by an inadequate production of NO,^33^ which lead us to believe that even with beta-blocking effects the therapy with nebivolol could be promising,^6^ but the results were not satisfactory.

Patients with HFNEF may present lower cardiac output during exercise, caused by an improper systolic volume due in large part to an impaired ventricular compliance.^27^ Peripheral O_2_ uptake is impaired in HFNEF, maybe due to intrinsic abnormalities in skeletal muscle cells or peripheral microcirculation function, compromising the patient's performance during the exercise.^27^ Therefore, all these factors leading to functional limitation in HFNEF should be therapeutic targets in this syndrome.^17^

Conraads et al.^29^ evaluated the therapy with nebivolol in HFNEF. They observed after 6 months with nebivolol a better control in SBP and HR at rest and peak effort but did not observe a positive impact in VO_2_, findings that are similar to those in our study. The authors^29^ attributed the lack of improvement in FC to chronotropic incompetence. In our study, we did not observe significant worsening in CRI after therapy, which justifies the chronotropic incompetence as the only factor responsible for the lack of nebivolol effect on the VO_2_. With 123I-MIBG scintigraphy, we can speculate that the factor responsible for the lack of a positive impact of nebivolol on FC is the absence of an effect on cardiac adrenergic activity, *i.e.,* the drug may not have acted effectively in one of the physiopathological pathways in HF.^25^ An adrenergic hyperactivity at rest can cause chronotropic incompetence during the exercise and, consequently, low FC.^14,32^

### Limitations

The main limitation of our study was the small number of patients. However, a calculation of the sample power showed that 25 patients would give sufficient statistical power to the study.

The lack of a placebo group and randomization were other limitations. The study was not randomized because we started the data collection from another study that was already in progress at our institution, but we respected the criterion for administration of the drug, in which the first 14 patients received treatment with nebivolol and the last 11 composed our control group.

Lack of a more detailed assessment of the occurrence of coronary disease was yet another limitation. However, in the absence of criteria to classify the HR etiology as ischemic and during the CPET, the absence of criteria do diagnose the patient with myocardial ischemia, we chose not to continue the investigation.

Finally, we can also cite as limitations the large number of obese individuals and short treatment duration. Obesity may have influenced our findings because obese patients may have low FC^34^ and adrenergic hypertonia.^35^ Despite the short treatment duration in our study, another study with HFREF published by Miranda et al.^36^ showed a positive response of carvedilol on 123I-MIBG uptake parameters after 3 months.

## Conclusion

Our findings suggest that even with a better control in SBP and HR at rest and peak effort and improvement in HRR, therapy with nebivolol was unable to promote a positive effect on FC and 123I-MIBG scintigraphic parameters. New studies using other strategies to improve cardiac adrenergic activity without impairing the HR response during exercise may be promising in patients with HFNEF.
